# Association of dietary fat intake and hepatocellular carcinoma among US adults

**DOI:** 10.1002/cam4.4256

**Published:** 2021-09-18

**Authors:** Iman Moussa, Rena S. Day, Ruosha Li, Ahmed Kaseb, Prasun K. Jalal, Carrie Daniel‐MacDougall, Rikita I. Hatia, Ahmed Abdelhakeem, Asif Rashid, Yun Shin Chun, Donghui Li, Manal M. Hassan

**Affiliations:** ^1^ Department of Epidemiology, Human Genetics, and Environmental Science School of Public Health The University of Texas Health Science Center at Houston Houston Texas USA; ^2^ Division of Epidemiology, Human Genetics, & Environmental Sciences Southwest Center for Occupational and Environmental Health Michael & Susan Dell Center for Healthy Living School of Public Health The University of Texas Health Science Center at Houston Houston Texas USA; ^3^ Department of Biostatistics and Data Science School of Public Health The University of Texas Health Science Center at Houston Houston Texas USA; ^4^ Department of Gastrointestinal Medical Oncology The University of Texas MD Anderson Cancer Center Houston Texas USA; ^5^ Division of Gastroenterology and Hepatology Department of Medicine Baylor College of Medicine Houston Texas USA; ^6^ Department of Epidemiology The University of Texas MD Anderson Cancer Center Houston Texas USA; ^7^ Department of Internal Medicine Baptist Hospitals of Southeast Texas Beaumont Texas USA; ^8^ Department of Pathology The University of Texas MD Anderson Cancer Center Houston Texas USA; ^9^ Division of Surgery Department of Surgical Oncology The University of Texas MD Anderson Cancer Center Houston Texas USA

**Keywords:** case–control, liver cancer, monounsaturated fatty acids, omega‐3 fatty acids, omega‐6 fatty acids, polyunsaturated fatty acids

## Abstract

**Background and Aims:**

The role of dietary fat consumption in the etiology of hepatocellular carcinoma (HCC) remains unclear. We investigated the associations of total fat and fatty acids with risk of HCC among US adults in a hospital‐based case–control study.

**Methods:**

We analyzed data from 641 cases and 1034 controls recruited at MD Anderson Cancer Center during 2001–2018. Cases were new patients with a pathologically or radiologically confirmed diagnosis of HCC; controls were cancer‐free spouses of patients with cancers other than gastrointestinal, lung, liver, or head and neck. Cases and controls were frequency‐matched by age and sex. Dietary intake was assessed using a validated food frequency questionnaire. Odds ratios (ORs) and corresponding confidence intervals (CIs) were computed using unconditional logistic regression with adjustment for major HCC risk factors, including hepatitis B virus and hepatitis C virus infection.

**Results:**

Monounsaturated fatty acid (MUFA) intake was inversely associated with HCC risk (highest vs. lowest tertile: OR, 0.49; 95% CI, 0.33–0.72). Total polyunsaturated fatty acid (PUFA) intake was directly associated with HCC risk (highest vs. lowest tertile: OR, 1.82; 95% CI, 1.23–2.70). Omega‐6 PUFA was directly associated with HCC risk (highest vs. lowest tertile: OR 2.29; 95% CI, 1.52–3.44). Long‐chain omega‐3 PUFA (eicosapentaenoic acid and docosahexaenoic acid) intake was also inversely associated with HCC risk (highest vs. lowest tertile: OR, 0.50; 95% CI, 0.33–0.70). No association was observed for saturated fat and HCC risk.

**Conclusion:**

Our findings support a direct association of omega‐6 PUFA intake with HCC and an inverse association of MUFA and long‐chain omega‐3 PUFA intake with HCC.

## Graphical abstract

We found hepatocellular carcinoma was positively associated with *n*‐6 polyunsaturated fatty acids intake, inversely associated with monounsaturated fatty acids and long‐chain omega‐3 fatty acids intake, and not associated with saturated fat intake.

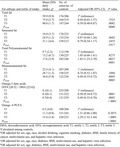

## 1 INTRODUCTION

Hepatocellular carcinoma (HCC) is the major type of primary liver cancer, and its incidence is increasing. It is the sixth most common cancer and the third leading cause of cancer‐related deaths in the world.^1^, ^2^ HCC advances rapidly and is usually diagnosed at late stages. The main etiologic factors of HCC are hepatitis B virus (HBV), hepatitis C virus (HCV), alcohol consumption, diabetes mellitus, cigarette smoking, and obesity.^3^, ^4^, ^5^, ^6^, ^7^ Approximately 20%–30% of HCC cases in the United States are attributed to obesity and diabetes.^8^ Common carcinogenic pathways of the different etiologies of HCC include chronic inflammation, continuous cycles of hepatic injury and regeneration resulting in malignant genetic alteration, and activation of oncogenes or suppression of tumor suppressor genes.^9^

Most risk factors for HCC, including HCV, diabetes, and obesity, are associated with accumulation of lipids in liver cells, known as fatty liver disease or hepatic steatosis. Concurrent with the increase in HCC incidence, hepatic steatosis is increasing in prevalence and becoming a growing global health problem.^10^, ^11^, ^12^ The liver plays a major role in lipid metabolism, and an increase in the intake of dietary fat, notably saturated fat, can lead to hepatic steatosis, which may progress to nonalcoholic steatohepatitis, an established risk factor for cirrhosis and HCC development.^13^, ^14^ Diet composition contributes to the development of obesity, diabetes, and steatosis; this has prompted the investigation of the role of diet in the development of HCC.

Although several different food components have been assessed with regard to HCC development, few studies have investigated the impact of a dietary fat and/or fatty acids on the risk of HCC; and overall, results for dietary risk factors have been inconsistent.^15^, ^16^, ^17^, ^18^, ^19^, ^20^, ^21^, ^22^, ^23^, ^24^, ^25^, ^26^, ^27^, ^28^, ^29^, ^30^ The large, prospective National Institutes of Health‐American Association of Retired Persons (NIH‐AARP) Diet and Health study showed a positive association between saturated fat intake and liver cancer.^19^ Another US cohort, the Nurses’ Health Study and the Health Professionals Follow‐up Study, reported an inverse association of monounsaturated fatty acids (MUFAs), *n*‐3 polyunsaturated fatty acids (PUFAs), and *n*‐6 PUFA with risk of HCC.^31^ However, these two US studies lacked information on HBV and HCV infection status, strong potential confounders; and two other studies conducted in Europe reported mixed results.^16^, ^23^

Given that the role of dietary fat in HCC etiology remains unclear, we conducted a case–control study to investigate (1) the association of dietary intake of total fat and major fatty acids including saturated fatty acids, MUFA, total PUFA, omega‐6 PUFA, and long‐chain omega‐3 PUFA [eicosapentaenoic acid (EPA) and docosahexaenoic acid (DHA)] with HCC risk among US patients with consideration of the effects of underlying viral infection and other etiological factors and (2) the potential interaction between dietary fat and other HCC risk factors.

## 2 MATERIALS AND METHODS

This investigation was part of an ongoing hospital‐based case–control study, which was approved by the Institutional Review Board at The University of Texas MD Anderson Cancer Center. Written informed consent for participation was obtained from each study participant. Subjects were recruited between 7 March 2001 and 5 March 2018. Patients newly diagnosed with HCC were prospectively enrolled at MD Anderson Cancer Center gastrointestinal medical oncology and surgical oncology outpatient clinics. Cases were included if they had a pathologically or radiologically confirmed diagnosis of HCC. Control subjects were cancer‐free spouses of patients with cancers other than gastrointestinal, lung, liver, or head and neck recruited from MD Anderson central diagnostic radiology clinics. A case could not have his/her spouse as a control. Cases and controls were US residents and were frequency‐matched by age (±5 years) and sex. We obtained 887 cases and 1093 controls with a completed food frequency questionnaire (FFQ). We excluded 52 cases with other types of primary liver cancer, such as cholangiocarcinoma and fibrolamellar HCC. We excluded 132 HCC cases with a prior history of cancer at other organs (67 cases with skin cancer were not excluded). An additional 6 cases and 17 controls were excluded for non‐US residency. We also excluded 56 cases and 42 controls with incomplete and/or improbably high or low total energy intake (>6000 kcal/day or <500 kcal/day). After these exclusions, 641 cases and 1034 controls remained in the final analysis. Participants’ weight history (for body mass index [BMI] calculation) was not collected from the beginning of the study, hence a total of 496 subjects (134 cases and 362 controls) did not have BMI data.

The definition and assessment of risk factors has been described in detail previously.^32^ At recruitment, cases and controls were interviewed in person using a validated structured questionnaire to collect information on HCC risk factors, including education, race, smoking, alcohol consumption, history of diabetes, medical history, family history of cancer, and height and weight history. Blood samples were collected from cases and controls and tested for HCV antibodies, hepatitis B surface antigen, and antibodies to hepatitis B core antigen. Cirrhosis diagnosis was abstracted from patients’ medical records. Patients were considered to have a history of cirrhosis if the medical record showed a corresponding pathologic finding (from diagnostic biopsy) or computed tomography scan or clinical signs of cirrhosis such as ascites, bleeding esophageal cancer, or hepatic encephalopathy.

The Willet semiquantitative FFQ was used to assess the usual dietary intake of participants during the past year (year prior to cancer diagnosis for cases and year prior to recruitment for controls).^33^ The validated FFQ queries the following categories: dietary supplements, dairy foods, fruits, vegetables, eggs and meat, breads and cereals, beverages, and sweets.^34^ The FFQ included standard portion sizes and frequency‐of‐consumption options ranging from “never, or less than once per month” to “≥6 per day” during the past year. Completed FFQs were processed by the Department of Nutrition at the Harvard T. H. Chan School of Public Health. The daily nutrient consumption was estimated using the Harvard School of Public Health’s nutrient database. Intake of dietary fat [saturated fat, MUFA, PUFA, omega‐6 PUFA, and long‐chain omega‐3 PUFA (EPA and DHA)] was adjusted for total energy intake using the residual method and categorized in tertiles based on the distribution in the controls.^35^

Multivariable logistic regression was used to compute odds ratios (ORs) and 95% confidence intervals (CIs) for the association between HCC and dietary fat intake. Age (<60 years and ≥60 years) and sex were included in every regression model. Selection of other variables for regression adjustment was based on the change in estimate approach.^36^ A covariate causing a 10% or greater change in the estimated OR for dietary fat intake was included in the final model. We evaluated the following risk factors for confounding: cigarette smoking (no smoking, ≤20 pack‐years of smoking, and >20 pack‐years of smoking), alcohol consumption (no drinking, ≤60 ml of ethanol per day, and >60 ml of ethanol per day), education level (less than a college education and college education and higher), race [non‐Hispanic White and other race (Hispanic, African American, and Asian)], family history of cancer (yes/no), diabetes (no diabetes, diabetes diagnosed ≤1 year of HCC diagnosis, and diabetes diagnosed >1 year of HCC diagnosis), mean BMI during early adulthood (mid‐20s to mid‐40s) [normal weight (≤24.9 kg/m^2^), overweight (25–29.9 kg/m^2^), and obese (≥30 kg/m^2^)], HCV/HBV infection (infection with either or both vs. none), and multivitamin use (yes vs. no). Test for linear trend was performed by entering the tertile scores of dietary fat intake as a continuous variable into the model.^37^ The population attributable risk percentage (PAR%) of HCC was calculated as follows: $\mathrm{PAR}\%=\frac{P_{e}\left(\text{OR}‐1\right)}{P_{e}\left(\text{OR}‐1\right)+1}\times 100$, in which OR is the adjusted OR for the relationship between dietary fat intake and having HCC and *P*_e_ is the prevalence of dietary fat consumption in the controls before enrollment.^38^

We stratified regression models by HCV/HBV infection, sex, and diabetes to assess for potential effect measure modification of the association between dietary fat intake and HCC risk due to these covariates. Given that cirrhosis is a primary risk factor for HCC and can lead to change in dietary habits, we examined the association of dietary fat subtype with cirrhosis among HCC cases (cases with vs. without cirrhosis). Additionally, sensitivity analyses were conducted to evaluate the association between dietary fat and HCC among patients without cirrhosis to address potential reverse causation.

We further examined the possible additive effect of dietary fat and HCV/HBV infection and of dietary fat and diabetes on HCC occurrence. We did this by (1) comparing the multivariable adjusted expected and observed joint effect of dietary fat type with HCV/HBV infection and dietary fat type with diabetes on HCC and (2) calculating synergy (or antagonism) index. Expected joint effect is the independent excess risk due to dietary fat plus the independent excess risk due to HCV/HBV infection. Synergy (or antagonism) index is the ratio of the observed joint effect to expected joint effect. An index <1 indicates antagonism, an index >1 indicates synergy, and an index equal to 1 indicates independent action of the risk factors on HCC occurrence. Potential multiplicative interaction between fat type and HCC risk factors (HCV/HBV infection and diabetes) was evaluated by including interaction terms formed by the product of the risk factor of interest and tertile of fat type in the logistic regression model.

All statistical analyses were completed using SAS, version 9.4 (SAS Institute, Inc.) with two‐sided tests. *p* < 0.05 was considered statistically significant.

## 3 RESULTS

Table 1 shows the distribution of HCC cases and controls by demographics and selected HCC risk factors. Most study subjects were non‐Hispanic White men; the male:female ratio was 2.8:1 among HCC cases. Compared to controls, HCC cases were more likely to be smokers, diabetic, obese, and heavy alcohol drinkers. Mean age (standard deviation) was 62.9 (10.9) years for cases and 60.0 (10.7) years for controls. As expected from previous studies,^32^, ^39^ HCV and/or HBV infection, obesity, diabetes, smoking, alcohol consumption, and family history of cancer were associated with increased risk of HCC.

**TABLE 1:** Multivariable adjusted ORs and 95% CIs for HCC for selected demographic and clinical characteristics

Characteristic	HCC patients (*n* = 487)^a^		Controls (*n* = 669)		Adjusted OR^b^ (95% CI)	*p* value
	*n*	%	*n*	%		
Sex						
Female	132	27.1	264	39.5	1 (reference)	
Male	355	72.9	405	60.5	0.97 (0.69–1.38)	0.8745
Age, years						
<60	176	36.1	294	44.0	1 (reference)	
≥60	311	63.9	375	56.0	2.65 (1.84–3.80)	<0.0001
Race						
Non‐Hispanic White	368	75.6	617	92.2	1 (reference)	
Another race	119	24.4	52	7.8	2.65 (1.63–4.30)	0.0001
Education						
<College education	190	39.0	173	25.9	1 (reference)	
≥College education	297	61.0	496	74.1	0.80 (0.57–1.12)	0.1912
Alcohol drinking						
No drinking	131	26.9	284	42.5	1 (reference)	
<60 ml ethanol/day	259	53.2	321	48.0	1.35 (0.96–1.89)	0.0879
≥60 ml ethanol/day	97	19.9	64	9.5	2.05 (1.26–3.34)	0.0040
Cigarette smoking						
No smoking	171	35.1	366	54.7	1 (reference)	
≤20 pack‐years	145	29.8	138	20.6	1.40 (0.94–2.08)	0.1002
>20 pack‐years	171	35.1	165	24.7	1.62 (1.12–2.35)	0.0106
Family history of cancer						
No	129	26.5	212	31.7	1 (reference)	
Yes	358	73.5	457	68.3	2.01 (1.41–2.87)	<0.0001
History of diabetes mellitus						
No diabetes	328	67.4	589	88.0	1 (reference)	
Diabetes ≤1 year of HCC diagnosis	8	1.6	15	2.2	1.20 (0.46–3.14)	0.7155
Diabetes >1 year of HCC diagnosis	151	31.0	65	9.7	3.83 (2.60–5.65)	<0.0001
BMI^c^						
Normal weight	290	59.6	439	65.6	1 (reference)	
Overweight	135	27.7	192	28.7	0.99 (0.69–1.42)	0.9522
Obese	62	12.7	38	5.7	2.92 (1.69–5.02)	0.0001
Hepatitis virus infection						
No virus infection	268	55.0	656	98.1	1 (reference)	
HCV and/or HBV infection	219	45.0	13	1.9	53.36 (28.49–99.94)	<0.0001
Multivitamin intake						
No	268	55.0	293	43.8	1 (reference)	
Yes	219	45.0	376	56.2	0.62 (0.45–0.84)	0.0026

Abbreviations: BMI, body mass index; CI, confidence interval; HBV, hepatitis B virus; HCC, hepatocellular carcinoma; HCV, hepatitis C virus; OR, odds ratio;

^a^ Missing data: Multivitamin intake (15 cases 2 controls); history of type 2 diabetes mellitus (1 case), hepatitis virus infection (4 cases), BMI (n= 134 cases and 362 controls); alcohol drinking (1 control).

^b^ The adjusted ORs were estimated from a multivariable logistic regression model adjusted for sex, age, race, education, alcohol drinking, cigarette smoking, diabetes, BMI, family history of cancer, and hepatitis virus infection.

^c^ Normal weight, ≤24.9 kg/m^2^; overweight, 25–29.9 kg/m^2^; obese, ≥30 kg/m^2^.

The adjusted ORs for HCC according to tertiles of energy‐adjusted total fat and fatty acid intake are shown in Table 2. Total fat intake was inversely associated with HCC risk; however, the association of fatty acid components and HCC varied. For MUFA, those in the highest tertile of intake had a 50% lower risk of HCC compared to those in the lowest tertile. For total PUFA, those in the highest tertile of intake had an 82% increased risk of HCC compared to those in the lowest tertile. For omega‐6 PUFA, those in the highest tertile of intake had more than twofold increased risk of HCC. The prevalence of omega‐6 PUFA consumption in the control population before enrollment (*P*_e_) was 223/669 = 0.33, and thus we estimated the PAR% for the highest tertile of omega‐6 PUFA intake for HCC as (0.33 x 1.29)/[(0.33 x 1.29) + 1] = 0.43/1.43 = 30%. For long‐chain omega‐3 fatty acids, those in the highest tertile of intake had a 50% lower risk of HCC compared to those in the lowest tertile. Excluding persons using omega‐3 supplements (13.7% of cases and 12.6% of controls) did not substantially change the OR estimate for long‐chain omega‐3 fatty acids intake (highest vs. lowest tertile of intake: OR, 0.48; 95% CI, 0.32–0.70; second vs. lowest tertile of intake: OR, 0.41; 95% CI, 0.27–0.62). Saturated fat intake was not associated with HCC.

**TABLE 2:** Multivariable adjusted ORs and 95% CIs for HCC according to tertiles of energy‐adjusted fat intake

Fat subtype and tertile of intake	Mean (SD) daily intake, g^a^	No. of cases/no. of controls	Adjusted OR (95% CI)	*p* value
Total fat				
T1	59.9 (9.9)	176/206	1^b^ (reference)	
T2	75.0 (2.7)	164/219	0.94 (0.65–1.37)	0.7525
T3	90.0 (1.7)	147/244	0.59 (0.40–0.87)	0.0082
*p*_trend_				0.0080
Saturated fat				
T1	18.9 (3.7)	176/223	1^b^ (reference)	
T2	24.9 (1.2)	153/234	0.87 (0.60–1.26)	0.4602
T3	31.1 (4.6)	158/212	0.79 (0.54–1.17)	0.2435
*p*_trend_				0.2417
Total polyunsaturated fat				
T1	9.7 (2.2)	112/198	1^b^ (reference)	
T2	13.2 (0.7)	130/225	1.05 (0.68–1.61)	0.8275
T3	17.6 (3.9)	245/246	1.82 (1.23–2.70)	0.0027
*p*_trend_				0.0001
Monounsaturated fat				
T1	22.4 (4.1)	207/208	1^c^ (reference)	
T2	28.7 (1.2)	158/225	0.74 (0.52–1.07)	0.1094
T3	36.0 (5.9)	122/236	0.49 (0.33–0.72)	0.0003
*p*_trend_				0.0003
Omega‐3 fatty acids (EPA [20:5] + DHA [22:6])				
T1	0.1 (0.1)	225/208	1^d^ (reference)	
T2	0.3 (0.1)	131/222	0.50 (0.34–0.72)	0.0001
T3	0.7 (0.4)	131/239	0.49 (0.33–0.70)	0.0002
*p*_trend_				<0.0001
Omega‐6 PUFA				
T1	6.5 (4.0)	108/204	1^b^ (reference)	
T2	11.3 (0.8)	151/242	1.31 (0.86–2.00)	0.2075
T3	15.4 (3.4)	228/223	2.29 (1.52–3.44)	<0.0001
*p*_trend_				<0.0001

Abbreviations: BMI, body mass index; CI, confidence interval; DHA, docosahexaenoic acid; EPA, eicosapentaenoic acid; HCC, hepatocellular carcinoma; OR, odds ratio; SD, standard deviation; T1, tertile 1; T2, tertile 2; T3, tertile 3.

^a^ Calculated among controls.

^b^ OR adjusted for sex, age, race, alcohol drinking, cigarette smoking, diabetes, BMI, family history of cancer, multivitamin use, and hepatitis virus infection.

^c^ OR adjusted for sex, age, race, diabetes, BMI, multivitamin use, and hepatitis virus infection.

^d^ OR adjusted for sex, age, diabetes, BMI, multivitamin use, and hepatitis virus infection.

The associations between dietary fat intake and HCC among subjects without cirrhosis are shown in Table 3. Results were similar to those observed in the entire study population. Examining the association between dietary fat intake and cirrhosis (regardless of the cause of cirrhosis) in patients with HCC (cirrhotic vs. noncirrhotic HCC) indicated that only intake of long‐chain omega‐3 fatty acids was significantly inversely associated with cirrhotic HCC (highest vs. lowest tertile of intake: OR, 0.54; 95% CI, 0.33–0.88).

**TABLE 3:** Multivariable adjusted ORs and 95% CIs for HCC according to tertiles of energy‐adjusted fat intake among HCC cases without cirrhosis

Fat subtype and tertile of intake	No. of cases/no. of controls	Adjusted OR^a^ (95% CI)	*p* value
Total fat			
T1	90/206	1 (reference)	
T2	82/219	0.95 (0.62–1.44)	0.9456
T3	76/244	0.73 (0.48–1.13)	0.1575
Saturated fat			
T1	98/223	1 (reference)	
T2	76/234	0.84 (0.56–1.27)	0.4046
T3	74/212	0.79 (0.52–1.21)	0.2751
Total polyunsaturated fat			
T1	60/198	1 (reference)	
T2	61/225	1.04 (0.64–1.68)	0.8883
T3	127/246	2.01 (1.30–3.11)	0.0018
Monounsaturated fat			
T1	106/208	1 (reference)	
T2	73/225	0.67 (0.44–1.01)	0.0585
T3	69/236	0.57 (0.37–0.87)	0.0095
Omega‐3 fatty acids (EPA [20:5] + DHA [22:6])			
T1	102/208	1 (reference)	
T2	69/222	0.59 (0.39–0.90)	0.0142
T3	77/239	0.58 (0.38–0.88)	0.0101
Omega‐6 polyunsaturated fat			
T1	59/204	1 (reference)	
T2	77/242	1.38 (0.85–2.22)	0.1896
T3	112/223	2.42 (1.52–3.85)	0.0002

Abbreviations: BMI, body mass index; CI, confidence interval; DHA, docosahexaenoic acid; EPA, eicosapentaenoic acid; HCC, hepatocellular carcinoma; OR, odds ratio; T1, tertile 1; T2, tertile 2; T3, tertile 3.

^a^ OR adjusted for sex, age, race, alcohol drinking, cigarette smoking, diabetes, BMI, family history of cancer, multivitamin use, and hepatitis virus infection.

The interaction indexes for dietary fat and HCV/HBV infection were 0.2 (95% CI, 0.15–0.23) for MUFA and 0.1 (95% CI, 0.02–0.53) for long‐chain omega‐3 fatty acids, indicating antagonism and possible attenuation of the HCC risk associated with HCV/HBV infection by MUFA and long‐chain omega‐3 fatty acid (Table 4). The interaction index for total PUFA and HCV/HBV infection was 0.9 (95% CI, 0.24–3.36), indicating no joint effect of PUFA and HCV/HBV on HCC risk. The interaction index for omega‐6 PUFA and HCV/HBV infection was 1.7 (0.43–6.62) indicating possible synergy between omega‐6 PUFA and HCV/HBV on HCC risk; however, it is not statistically significant. Results of further analysis of multiplicative interaction between HCV/HBV infection and dietary fat subtype intake were not significant (data not shown).

**TABLE 4:** Multivariable adjusted ORs and 95% CIs for HCC according to dietary fat intake, HCV/HBV infection, and diabetes with interaction indexes

Fat subtype and tertile of intake	HCV/HBV	Cases/controls	AOR (95% CI)	Diabetes	Cases/controls	AOR (95% CI)
Polyunsaturated fat						
T1	No	55/194	1^a^	No	89/181	1^b^
T1	Yes	57/4	68.64 (22.62–208.25)	Yes	23/17	2.44 (1.07–5.56)
T3	No	149/240	1.93 (1.28–2.91)	No	149/211	1.66 (1.07–2.57)
T3	Yes	96/6	61.81 (24.36–156.86)	Yes	96/35	6.35 (3.61–11.18)
Expected joint effect	68.57			2.1		
Interaction index (95% CI) 0.9 (0.24–3.36)				3.0 (1.3–7.2)		
Monounsaturated fat						
T1	No	107/204	1^a^	No	161/186	1^b^
T1	Yes	100/4	69.21 (23.42–204.57)	Yes	46/22	2.88 (1.50–5.52)
T3	No	74/228	0.50 (0.33–0.75)	No	66/199	0.48 (0.30–0.74)
T3	Yes	48/8	11.98 (5.01–28.71)	Yes	56/37	1.46 (0.79–2.68)
Expected joint effect	67.71			1.36		
Interaction index (95% CI) 0.2 (0.15–0.23)				1.1 (0.20–5.92)		
Omega‐3 fatty acids						
T1	No	125/206	1^a^	No	160/178	1^b^
T1	Yes	100/2	134.44 (30.99–583.25)	Yes	65/30	2.28 (1.27–4.08)
T3	No	75/232	0.54 (0.36–0.80)	No	84/208	0.45 (0.29–0.70)
T3	Yes	56/7	16.40 (6.69–40.21)	Yes	47/31	1.40 (0.74–2.63)
Expected joint effect	132.98			0.73		
Interaction index (95% CI) 0.1 (0.02–0.53)				1.9 (0.24–15.5)		
Omega‐6 PUFA						
T1	No	51/199	1^a^	No	84/183	1^b^
T1	Yes	57/5	62.43 (22.39–174.06)	Yes	24/21	2.24 (1.01–4.97)
T3	No	134/219	2.34 (1.52–3.59)	No	144/197	1.97 (1.25–3.12)
T3	Yes	94/4	105.59 (35.31–315.76)	Yes	84/26	8.76 (4.68–16.39)
Expected joint effect	62.77			2.21		
Interaction index (95% CI) 1.7 (0.43–6.62)				4.0 (3.1–9.0)		

Abbreviations: AOR, multivariable adjusted OR; BMI, body mass index; CI, confidence interval; HBV, hepatitis B virus; HCC, hepatocellular carcinoma; HCV, hepatitis C virus; PUFA, polyunsaturated fatty acid; OR, odds ratio; T1, tertile 1; T2, tertile 2; T3, tertile 3.

^a^ OR adjusted for sex, age, race, alcohol drinking, cigarette smoking, diabetes, BMI, family history of cancer, and multivitamin use.

^b^ OR adjusted for sex, age, race, alcohol drinking, cigarette smoking, BMI, family history of cancer, multivitamin use, and hepatitis virus infection.

Evaluation of the joint effect of dietary fat and diabetes on HCC risk showed that the joint effect of total PUFA and diabetes, also omega‐6 PUFA and diabetes was statistically significant (Table 4). Compared to subjects without diabetes and with the lowest tertile of omega‐6 PUFA intake, subjects with diabetes and highest omega‐6 PUFA intake had eight times the risk of HCC, and the synergy index was 4.0 (95% CI, 4.68–16.39), suggesting a positive additive effect of high omega‐6 PUFA intake with diabetes on HCC. In other words, the combined effect of diabetes and high omega‐6 PUFA intake could be associated with HCC risk higher than we would expect from the sum of the individual effects of these two risk factors. The test for multiplicative interaction between diabetes and fat subtypes was not significant for PUFA (data not shown).

Stratified analysis showed that the positive association between total PUFA and HCC (highest vs. lowest tertile: OR, 1.84; 95% CI, 1.22–2.77), omega‐6 PUFA and HCC (highest vs. lowest tertile: OR, 2.23; 95% CI, 1.46–3.43), and the inverse association between long‐chain omega‐3 fatty acids and HCC (highest vs. lowest tertile: OR, 0.52; 95% CI, 0.35–0.76; second vs. lowest tertile: OR, 0.49; 95% CI, 0.33–0.72) persisted among subjects without HCV/HBV infection. The inverse association between MUFA and HCC also persisted among subjects without HCV/HBV infection, though it was not statistically significant (highest vs. lowest tertile: OR, 0.77; 95% CI, 0.52–1.15). Among subjects with HCV/HBV infection, the associations between HCC and different fat subtypes were not significant, likely because of the small number of controls with HCV/HBV infection. Furthermore, stratified analysis showed that the association between dietary fat intake and HCC differed by sex and diabetes (Table S1). The positive association between total PUFA, omega‐6 PUFA intake, and HCC seemed to be restricted to males. However, the inverse associations of MUFA and long‐chain omega‐3 fatty acids with HCC were similar among men and women. The magnitude of the association between increased omega‐6 PUFA intake and HCC was stronger among subjects with diabetes than among those without; HCC risk was four times as high among individuals in the highest tertile as it was among those in the lowest tertile. Excluding those who had diabetes diagnosed within a year of HCC diagnosis did not change the results. The associations between MUFA and long‐chain omega‐3 fatty acids and HCC did not differ by diabetes status.

## 4 DISCUSSION

In this case–control study, we found a direct association of total PUFA intake and HCC, mainly driven by omega‐6 PUFA intake and inverse associations of MUFA and long‐chain omega‐3 fatty acids (EPA and DHA) intake with HCC. Saturated fat intake was not associated with HCC. In stratified analyses, we found that the association of total PUFA, omega‐6 PUFA intake with HCC was more pronounced among males than females and among diabetics than nondiabetics. Our results indicated that approximately 30% of the HCC cases in our study could be explained by high omega‐6 PUFA intake after consideration of other known risk factors, most notably HCV/HBV infection.

Few observational studies have explored the association between dietary fat and HCC, and the results of those studies are inconsistent. Similar to our findings, a cohort analysis using the Nurses’ Health Study and the Health Professionals Follow‐up Study, showed inverse association of MUFA and *n*‐3 PUFA with risk of HCC; however, in contrast to our findings this study reported an inverse association of *n*‐6 PUFA with HCC.^31^ However, that study did not adjust for confounding by HCV/HBV infection. The European Prospective Investigation into Cancer showed negative associations of MUFA intake with HCC and fish intake (long‐chain omega‐3 fatty acid source) with HCC and no association of PUFA or saturated fats with HCC.^16^ Consistent with our findings, the Singapore Chinese Health Study showed a positive association of omega‐6 PUFA intake with HCC risk; it also showed a suggestive inverse association of MUFA intake with HCC risk.^40^ Furthermore, our results were consistent with a meta‐analysis showing a lower risk of HCC in the highest versus lowest category of omega‐3 fatty acid intake.^41^ The NIH‐AARP Diet and Health study showed a positive association for saturated fat intake, no association for PUFA, and a non‐significant association for MUFA intake with HCC; however, that study did not include HCV/HBV infection status as a potential confounder.^19^ Also contrasting with our results, an Italian hospital‐based case–control study showed a negative association between PUFA and HCC and no association of MUFA with HCC.^23^, ^26^

We further observed an additive effect of omega‐6 PUFA and diabetes on HCC, but an antagonistic effect of diabetes and MUFA or long‐chain omega‐3 fatty acids on HCC. These results suggest that among diabetic individuals, omega‐6 PUFA might facilitate the progression of liver disease to HCC, while a diet higher in long‐chain omega‐3 fatty acids and/or MUFA might reduce the odds of HCC. The Singapore Chinese Health Study similarly reported an increased risk of HCC with *n*‐6 PUFA dietary intake stronger among diabetic individuals (for the highest quartile vs. the lowest: hazard ratio [HR], 1.88; 95% CI, 0.86–4.09) than among non‐diabetic individuals (for the highest quartile vs. the lowest: HR, 1.4; 95% CI, 0.97–2.01), although the difference was not statistically significant.^40^

There are several possible mechanisms whereby dietary fat may be associated with HCC risk. Dietary fat might be associated with factors that predispose to HCC, such as insulin resistance, inflammation, and nonalcoholic fatty liver disease.^42^ Different fatty acids comprising dietary fat components have different roles in cell growth, immune response, and tumor growth. Few human studies have replicated findings for dietary fat and cancer observed in experimental models. The concept that dietary fat plays a role in cancer development is largely based on experimental studies; the findings of epidemiological studies have been controversial. In animal models, diets high in saturated fatty acids promoted breast, colorectal, and prostate cancer, and diets high in *n*‐3 PUFA, particularly EPA and DHA, inhibited breast and colon tumor growth and metastasis.^43^ The effect of *n*‐3 and *n*‐6 PUFA on cancer risk had been conflicting^44^; *n*‐3 PUFA might be protective while *n*‐6 PUFA might induce cancer progression.^45^ The *n*‐6 PUFA arachidonic acid is a precursor of pro‐inflammatory eicosanoids with carcinogenic effects.^46^ The products of *n*‐6 PUFA peroxidation such as epoxides and aldehydes have been found to enhance tumor formation via oxidative DNA damage, whereas *n*‐3 PUFA are thought to increase the accumulation of lipid peroxidation products in tumor cells, inhibiting their growth.^47^, ^48^ Dietary lipids could exert carcinogenic effects by modulating the immune system; *n*‐6 PUFA promotes the formation of inflammatory cytokines such as tumor necrosis factor‐α (TNF‐α) and interleukin‐6 (IL‐6); however, *n*‐3 PUFA in the diet decreases the release of inflammatory cytokines such as TNF‐α and IL‐1β. We hypothesize that *n*‐3 and *n*‐6 PUFA effect on HCC risk could be driven by their effect on nonalcoholic steatohepatitis pathogenesis; a simple reversible steatosis can progress to nonalcoholic steatohepatitis, characterized by chronic cellular stress, liver inflammation (steatohepatitis), fibrosis, and a substantial increase in cirrhosis‐induced HCC risk.^49^, ^50^, ^51^

This study is limited by the recall bias inherent to case–control studies as cases might recall their usual dietary habits differently than healthy controls. To address recall bias, we assessed dietary intake over the year prior to HCC diagnosis for cases and the year prior to recruitment for controls so measurement error would most likely be non‐differential with respect to disease status. Also, reverse causation is possible because patients could alter their dietary habits just prior to or following disease diagnosis. To address reverse causation, we repeated the analysis among cases without cirrhosis. The rationale for this separate analysis is that cirrhosis usually precedes HCC and might cause patients to alter their dietary habits. Our findings among subjects without cirrhosis were similar to those in the total study population, indicating low potential of reverse causation. Another limitation of this study is the missing BMI information. Participants’ height and weight were not collected from the beginning of the study, and 496 subjects (134 cases and 362 controls) did not have BMI data, limiting use of this measure. However, the demographic characteristics and dietary intake of participants missing BMI data did not differ from those of participants with BMI data. The results of this and other stratification analysis should be interpreted with caution given the small sample size of the strata.

This study used self‐report to obtain information about several nondietary risk factors, including education, smoking, drinking habits, and diabetes. We previously indicated that self‐reported information about HCC risk factors in this study population was consistent with information obtained from patient medical records; thus, misreporting is assumed to be minimal.^39^ The reported associations between HCC and these factors from our case–control study, such as the association between HCC risk and alcohol drinking and cigarette smoking, were consistent with previously published results from population‐based studies. Moreover, agreement between self‐reported disease diagnosis and medical conditions has previously been documented,^52^, ^53^ which supports the reliability and validity of self‐reports of diabetes mellitus. In addition, several studies showed high correlations between recalled and measured weight and height in young adulthood among middle‐aged and older men and women.^54^, ^55^, ^56^, ^57^

We did not adjust directly for cirrhosis in our study. Cirrhosis is considered end‐stage chronic liver disease, diagnosed pathologically or radiologically and patients with cirrhosis are seriously ill with multiple symptoms. We believe that the prevalence of cirrhosis in the healthy controls of the study is extremely low and will be difficult to assess in all controls by biopsy or radiology. However, we previously showed that alcohol, HCV/HBV infection, diabetes, and obesity are major risk factors of cirrhosis.^32^, ^39^, ^58^, ^59^, ^60^ Accordingly, we adjusted for these factors as surrogates of cirrhosis.

Selection bias could result from recruiting hospital‐based cases with advanced‐stage HCC; however, selection bias of cases and controls is unlikely in this study for several reasons. First, HCC is often detected at late stages, and similarly, around 70% of our cases had advanced‐stage HCC. Second, only US cases and controls were included, and they had similar patterns of geographical residence. Third, controls were spouses of patients with non‐HCC cancers. Fourth, the dietary fat consumption for the controls was consistent with the dietary fat consumption for US adults shown by the National Health and Nutrition Examination Survey (2015–2016).

Last, the small number of non‐White controls limited the analysis of the association between HCC and energy‐adjusted dietary fat intake across racial groups. We compared the mean dietary fat intake of HCC cases to all study controls by race. Seventy percent of Hispanic and 65.4% of African American cases had a mean intake of long‐chain omega‐3 fatty acids lower than that of controls. Also, 57.1% of Hispanic and 75% of African American cases had a mean intake of MUFA lower than that of controls. Approximately half of the cases, of all races, had a higher mean intake of PUFA than that of controls.

Despite our study’s limitations, a hospital‐based study design might be the ideal epidemiological approach to study HCC and had many notable strengths. This study is one of the largest case–control studies of HCC among US adults with dietary and nondietary HCC risk factors, which facilitated adjustment for a wide range of potential confounders, including education, family history of cancer, BMI, smoking, alcohol consumption, diabetes, and infection with HCV and HBV. Previous studies on HCC and diet lacked data on HCV/HBV infection and therefore did not adjust for HCV/HBV infection and did not report their findings among non‐infected persons.^19^, ^31^, ^61^ HCC and cirrhosis diagnosis were pathologically or radiologically confirmed to avoid misdiagnosis of disease.

In conclusion, this large study provided evidence of a positive association between total PUFA and omega‐6 PUFA intake and HCC and inverse associations between long‐chain omega‐3‐fatty acids and MUFA intake and HCC among US adults. Our findings may have important clinical implications, we observed that high dietary intake of MUFA and long‐chain omega‐3 fatty acids attenuated the odds of HCC due to HCV/HBV infection. Thus, consuming diets high in long‐chain omega‐3‐fatty acids and MUFA might reduce the risk of HCC after treatment with direct‐acting or reduce HCV relapse or treatment failure. While our findings seem promising, replicating these findings in prospective and racially diverse cohort studies with more African Americans and Hispanics will help in developing dietary recommendations to decrease the incidence of HCC. Also, considering the low incidence and poor prognosis of HCC and the high incidence of fatty liver disease, the role of dietary fat in HCC prevention should be investigated in future dietary intervention studies among individuals at high risk for HCC.

## CONFLICT OF INTEREST

The authors disclose no conflict of interest.

## Supporting information

Table S1

## Data Availability

The data that support the findings of this study are available from the corresponding author upon reasonable request.
