# Supplementary material for: Association of dietary fat intake and hepatocellular carcinoma among US adults
Source: Cancer Med. 2021 Sep 18;10(20):7308–19. doi: 10.1002/cam4.4256 (PMC8525131; doi:10.1002/cam4.4256)
Supplement: Supplementary file 1 — Table S1 [file CAM4-10-7308-s001.docx]

**Supplemental Table 1**. Multivariable adjusted ORs and 95% CIs for HCC according to tertiles of energy-adjusted fat intake, stratified by sex and diabetes

|  | **Males** | | |  |  | **Females** | |
| --- | --- | --- | --- | --- | --- | --- | --- |
| Fat subtype  and tertile of intake | Cases/  Controls | AOR (95% CI) | *P* value |  | Cases/  Controls | AOR  (95% CI) | *P* value |
| Polyunsaturated fat |  |  |  |  |  |  |  |
| T1 | 80/141 | 1^a^ (reference) |  |  | 32/57 | 1 (reference) |  |
| T2 | 95/122 | 1.18 (0.69-2.01) | .5470 |  | 35/103 | 0.69 (0.32-1.46) | .3319 |
| T3 | 180/142 | 2.11 (1.31-3.42) | .0023 |  | 65/104 | 1.04 (0.52-2.10) | .9065 |
| Monounsaturated fat |  |  |  |  |  |  |  |
| T1 | 141/131 | 1^a^ (reference) |  |  | 66/77 | 1 (reference) |  |
| T2 | 119/125 | 0.85 (0.53-1.35) | .4821 |  | 39/100 | 0.62 (0.35-1.10) | .1039 |
| T3 | 95/149 | 0.54 (0.34-0.87) | .0116 |  | 27/87 | 0.40 (0.20-0.77) | .0059 |
| Omega-3 fatty acids  (EPA [20:5]  + DHA [22:6]) |  |  |  |  |  |  |  |
| T1 | 169/126 | 1^a^ (reference) |  |  | 56/82 | 1 (reference) |  |
| T2 | 88/136 | 0.41 (0.25-0.66) | .0002 |  | 43/86 | 0.71 (0.39-1.27) | .2464 |
| T3 | 98/143 | 0.52 (0.33-0.82) | .0055 |  | 33/96 | 0.45 (0.24-0.84) | .0121 |
| Omega-6 PUFA |  |  |  |  |  |  |  |
| T1 | 78/142 | 1^a^ (reference) |  |  | 30/62 | 1 (reference) |  |
| T2 | 106/143 | 1.36 (0.81-2.29) | .8391 |  | 45/99 | 0.93 (0.44-1.95) | .2456 |
| T3 | 171/120 | 2.74 (1.65-4.53) | .5029 |  | 57/103 | 1.27 (0.63-2.57) | .0001 |
|  | | **Diabetes** | |  |  | **No Diabetes** | |
| Polyunsaturated fat |  |  |  |  |  |  |  |
| T1 | 23/17 | 1^b^ (reference) |  |  | 89/181 | 1 (reference) |  |
| T2 | 40/28 | 1.69 (0.57-5.0) | .3421 |  | 90/197 | 1.09 (0.67-1.76) | .7406 |
| T3 | 96/35 | 3.22 (1.21-8.61) | .0195 |  | 149/211 | 1.78 (1.14-2.78) | .0116 |
| Monounsaturated fat |  |  |  |  |  |  |  |
| T1 | 46/22 | 1^b^ (reference) |  |  | 161/186 | 1 (reference) |  |
| T2 | 57/21 | 1.19 (0.52-2.73) | .6771 |  | 101/204 | 0.68 (0.45-1.02) | .0628 |
| T3 | 56/37 | 0.50 (0.23-1.10) | .0862 |  | 66/199 | 0.53 (0.34-0.83) | .005 |
| Omega-3 fatty acids  (EPA [20:5] + DHA [22:6]) |  |  |  |  |  |  |  |
| T1 | 65/30 | 1^b^ (reference) |  |  | 160/178 | 1 (reference) |  |
| T2 | 47/19 | 0.87 (0.39-1.91) | .7245 |  | 84/203 | 0.44 (0.29-0.67) | .0001 |
| T3 | 47/31 | 0.53 (0.25-1.12) | .0946 |  | 84/208 | 0.48 (0.32-0.74) | .0009 |
| Omega-6 PUFA |  |  |  |  |  |  |  |
| T1 | 24/21 | 1(reference) |  |  | 84/183 | 1(reference) |  |
| T2 | 51/33 | 1.66 (0.61-4.51) | 0.3169 |  | 100/209 | 1.29 (0.80-2.08) | 0.3048 |
| T3 | 84/26 | 4.35 (1.62-11.70) | 0.0036 |  | 144/197 | 2.13(1.34-3.38) | 0.0014 |

AOR, multivariable adjusted OR DHA, docosahexaenoic acid; EPA, eicosapentaenoic acid; T1, tertile 1; T2, tertile

^a^OR adjusted for age, race, alcohol drinking, cigarette smoking, diabetes, BMI, family history of cancer, multivitamin use, and hepatitis virus infection.

^b^OR adjusted for sex, age, race, alcohol drinking, cigarette smoking, BMI, family history of cancer, multivitamin use, and hepatitis virus infection.
